# Antagonising Wnt/β-catenin signalling ameliorates lens-capsulotomy-induced retinal degeneration in a mouse model of diabetes

**DOI:** 10.1007/s00125-018-4682-3

**Published:** 2018-07-17

**Authors:** Jose R. Hombrebueno, Imran H. A. Ali, Jian-xing Ma, Mei Chen, Heping Xu

**Affiliations:** 1 Centre for Experimental Medicine, Wellcome-Wolfson Institute of Experimental Medicine, School of Medicine, Dentistry and Biomedical Sciences, Queen’s University Belfast, 97 Lisburn Road, Belfast, BT9 7BL UK; 2 Department of Physiology, Harold Hamm Diabetes Center, University of Oklahoma Health Sciences Center, Oklahoma City, OK USA

**Keywords:** Cataract surgery, Diabetic retinopathy, Electroretinography, Inflammation, *Ins2*^*Akita*^, Neurodegeneration, SD-OCT

## Abstract

**Aims/hypothesis:**

Cataract surgery in diabetic individuals worsens pre-existing retinopathy and triggers the development of diabetic ocular complications, although the underlying cellular and molecular pathophysiology remains elusive. We hypothesise that lens surgery may exaggerate pre-existing retinal inflammation in diabetes, which may accelerate neurovascular degeneration in diabetic eyes.

**Methods:**

Male heterozygous *Ins2*^*Akita*^ mice (3 months of age) and C57BL/6 J age-matched siblings received either lens capsulotomy (to mimic human cataract surgery) or corneal incision (sham surgery) in the right eye. At different days post surgery, inflammation in anterior/posterior ocular tissues was assessed by immunohistochemistry and proinflammatory gene expression in the retina by quantitative PCR (qPCR). Degenerative changes in the retina were evaluated by electroretinography, in vivo examination of retinal thickness (using spectral domain optical coherence tomography [SD-OCT]) and morphometric analysis of retinal neurons. The therapeutic benefit of neutralising Wnt/β-catenin signalling following lens capsulotomy was evaluated by intravitreal administration of monoclonal antibody against the co-receptor low-density lipoprotein receptor-related protein 6 (LRP6) (Mab2F1; 5 μg/μl in each eye).

**Results:**

Lens capsulotomy triggered the early onset of retinal neurodegeneration in *Ins2*^*Akita*^ mice, evidenced by abnormal scotopic a- and b-wave responses, reduced retinal thickness and degeneration of outer/inner retinal neurons. Diabetic *Ins2*^*Akita*^ mice also had a higher number of infiltrating ionised calcium-binding adapter molecule 1 (IBA1)/CD68^+^ cells in the anterior/posterior ocular tissues and increased retinal expression of inflammatory mediators (chemokine [C-C motif] ligand 2 [CCL2] and IL-1β). The expression of β-catenin was significantly increased in the inner nuclear layer, ganglion cells and infiltrating immune cells in *Ins2*^*Akita*^ mice receiving capsulotomy. Neutralisation of Wnt/β-catenin signalling by Mab2F1 ameliorated ocular inflammation and prevented capsulotomy-induced retinal degeneration in the *Ins2*^*Akita*^ mouse model of diabetes.

**Conclusions/interpretation:**

Targeting the canonical Wnt/β-catenin signalling pathway may provide a novel approach for the postoperative management of diabetic individuals needing cataract surgery.

**Electronic supplementary material:**

The online version of this article (10.1007/s00125-018-4682-3) contains peer-reviewed but unedited supplementary material, which is available to authorised users.

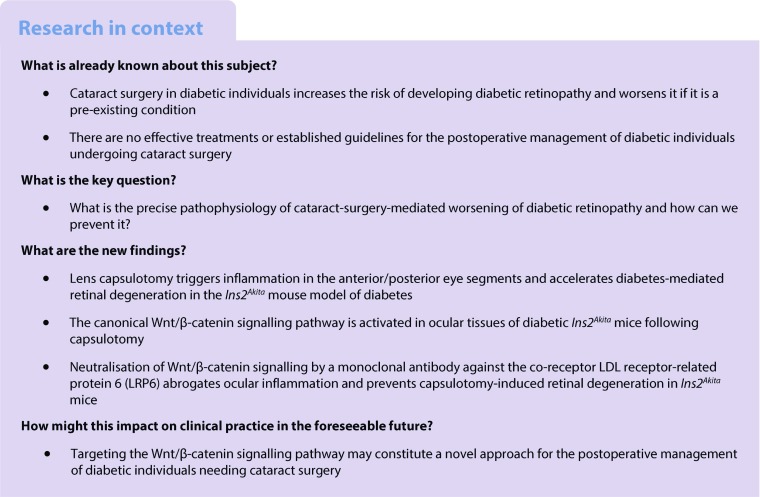



## Introduction

With an ageing population and the growing prevalence of obesity due to increasingly sedentary lifestyles, the incidence of diabetes mellitus is rising at an alarming rate [1]. One of the common complications associated with diabetes is diabetic retinopathy, which is the leading cause of vision loss in the working-age population [2]. Diabetic retinopathy is a progressive disorder that results in alterations in the neuroretina, leading to retinal neurodegeneration and microangiopathy in its early stages [3]. Non-proliferative diabetic retinopathy can lead to retinal non-perfusion, which ultimately drives pathological intraocular neovascularisation, known as proliferative diabetic retinopathy [4]. How diabetes leads to diabetic retinopathy is still not fully understood; however, inflammation is known to play an important role in its development and progression [5]. Other ocular complications associated with diabetes include cataract and corneal diseases [6]. Cataract is the leading cause of blindness worldwide and one of the major factors responsible for vision impairment [7].

Clinical studies have shown a clear link between diabetes and cataract formation [8]. Diabetic individuals tend to develop cataracts prematurely and progression is more rapid compared with people without diabetes [9, 10]. Owing to the rising prevalence of diabetes, the incidence of diabetic cataracts is also increasing [10]. Although cataract surgery is a routine procedure, it is not without risk. There is mounting evidence linking the development and/or exacerbation of pre-existing diabetic retinopathy following cataract surgery. Individuals who already have pre-existing active proliferative retinopathy and/or macular oedema tend to have the poorest outcomes following cataract surgery, such as a significant decrease in visual acuity and fast progression of diabetic retinopathy [11]. Phacoemulsification, the most common clinical procedure for cataract surgery, is shown to accelerate the progression of diabetic retinopathy in 34–50% of individuals [12–14], and induces macular oedema in 25–55% of diabetic individuals [15, 16]. The molecular mechanism underlying cataract-surgery-mediated worsening of diabetic retinopathy remains poorly defined, resulting in the absence of established guidelines for this ocular complication.

Previous investigations suggest a link between cataract surgery and exacerbation of ocular inflammation [17, 18]. For example, extracapsular lens extraction is shown to upregulate the expression of proinflammatory genes in the murine retina, including *Ccl2* and *Il-1β* (also known as *Il1b*) [19]. This can be problematic when considering the immunoprivileged status of the retina, as inflammatory mediators may actively drive retinal neurodegeneration [20]. Nevertheless, it remains unclear whether cataract-surgery-mediated worsening of diabetic retinopathy is elicited by exacerbation of retinal inflammation.

Activation of the canonical Wnt/β-catenin signalling pathway has been previously reported in diabetic retinopathy and its associated complications [21]. Besides its well-known role in embryonic development, Wnt/β-catenin activation is known to exert proinflammatory functions [22, 23]. For example, retinal microglia treated with recombinant Wnt3A (ligand for the canonical Wnt pathway) induced the expression and release of proinflammatory cytokines, including IL-6, IL-12 and TNF-α [22], demonstrating a regulatory role for Wnt signalling in retinal macrophage activation.

Extracapsular lens extraction is rarely performed in diabetic individuals. Phacoemulsification and intraocular lens implantation cannot be performed in mouse eyes. In this study, we performed lens capsulotomy to mimic two components of human cataract surgery in a murine model of type 1 diabetes (*Ins2*^*Akita*^ mouse): (1) a surgery at the anterior part of the eye with minimal traumatic insults; and (2) the release of lens protein into the anterior chamber of the eye. We then investigated the associated ocular immune and retinal degenerative pathogenesis.

## Methods

### Animals

Male heterozygous *Ins2*^*Akita*^ mice of C57BL/6 J background (C57BL/6-*Ins2*^*Akita*^/J, Jackson Laboratory, Bar Harbor, ME, USA) and non-diabetic siblings were used. The *Ins2*^*Akita*^ mice develop severe hyperglycaemia (>13.9 mmol/l) by 4 weeks of age [24]. *Cx3cr1*^GFP/GFP^ and C57BL/6 J mice were bred to obtain heterozygous *Cx3cr1*^+/GFP^ mice (which show green fluorescent protein [GFP]^+^ monocyte-derived cells but not chemokine [C-X3-C motif] receptor 1 [CX3CR1] deficiency). Mice were housed in standard pathogen-free animal housing rooms with 12/12 h light/dark cycle and free access to food and water. All procedures were conducted following the Guide for the Care and Use of Laboratory Animals and the regulation of the UK Home Office Animals (Scientific Procedures) Act 1986. Animal experiments followed the guidelines of the Association for Research in Vision and Ophthalmology (ARVO) on the use of animals in ophthalmic and vision research.

### Lens capsulotomy surgery

The traditional cataract surgery, extracapsular lens extraction, induces rapid posterior capsule opacification in mice [25], which prevents further investigation of diabetic retinopathy. We therefore conducted lens capsulotomy in 3-month-old mice (this age was kept constant for all experimental groups and strains across the study) to mimic human cataract surgery. Mice were anaesthetised by isoflurane and pupils dilated with 1% (wt/vol.) tropicamide and 2.5% (wt/vol.) phenylephrine (Laboratoire Chauvin, Montpellier, France).

**Fig. 1:**
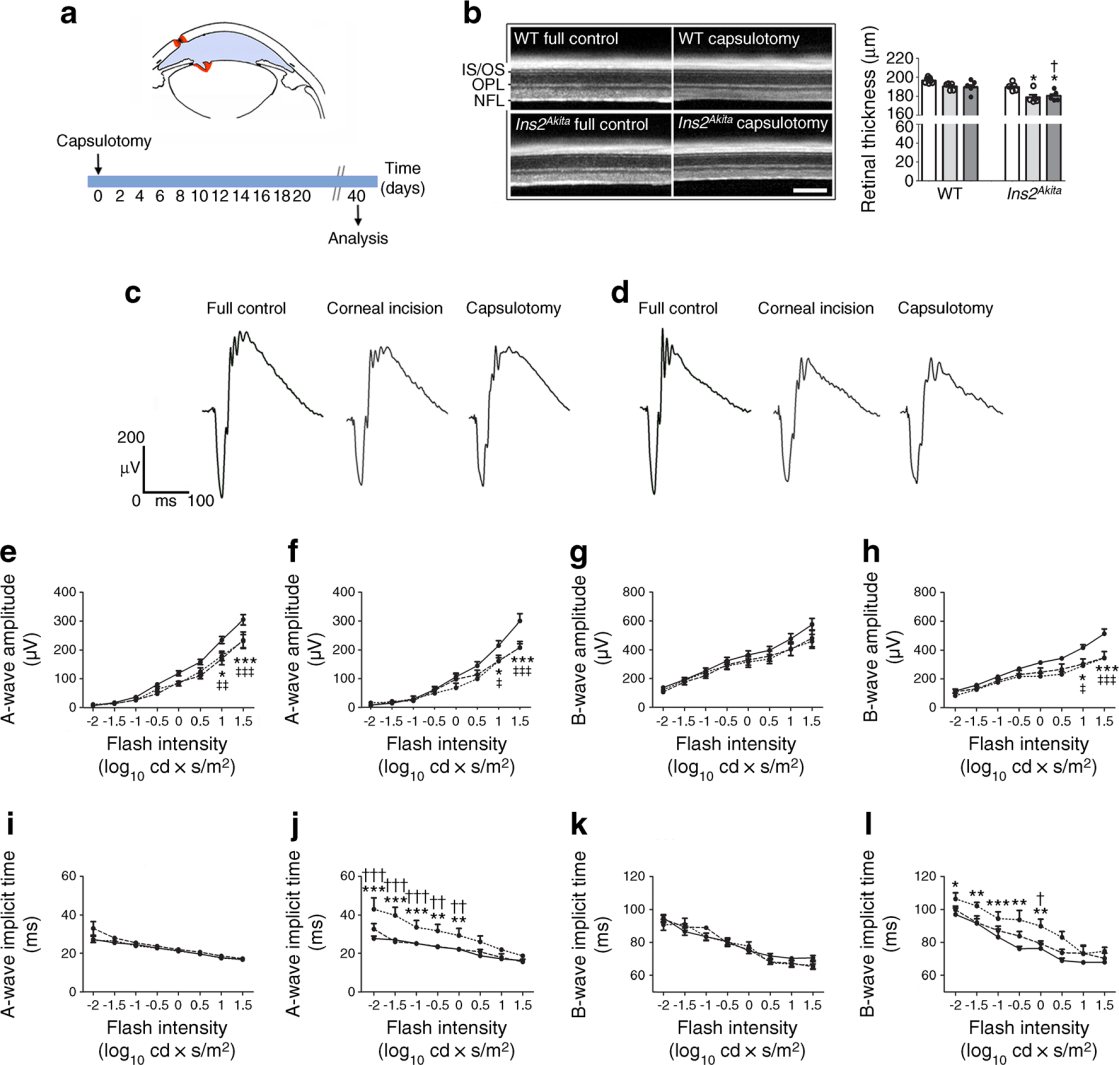
Retinal degeneration and abnormal scotopic ERG responses following capsulotomy. (**a**) Three-month-old WT and *Ins2*^*Akita*^ mice underwent corneal incision or lens capsulotomy and eyes were assessed 40 days after surgery for retinal degeneration. The red areas on the image show the positions where corneal incision and capsulotomy were performed (for a more detailed schematic see ESM Fig. 1). (**b**) Representative SD-OCT images from WT or *Ins2*^*Akita*^ mice following corneal incision or capsulotomy. The quantitative analysis of neuroretinal thickness by SD-OCT (from the photoreceptor inner segments/outer segments [IS/OS] to NFL) highlights significant retinal degeneration resulting from ocular surgery in *Ins2*^*Akita*^ mice. White bars, full control; light grey bars, corneal incision; dark grey bars, capsulotomy. (**c**, **d**) Representative scotopic ERG responses from WT (**c**) and *Ins2*^*Akita*^ (**d**) mice of different treatment groups, as indicated. (**e**–**l**) The amplitude (μV) and implicit time (ms) of a-waves and b-waves in WT (**e**, **g**, **i**, **k**) and *Ins2*^*Akita*^ (**f**, **h**, **j**, **l**) mice (*n* = 7 mice for WT full controls; *n* = 5 mice for WT and *Ins2*^*Akita*^ remaining groups). ERG white light flashes are presented as log_10_ cd × s/m^2^. In (**e**–**l**): solid line, full control; dashed line, corneal incision; dotted line, capsulotomy. Results are presented as mean ± SEM. *p < 0.05, **p < 0.01 and ***p < 0.001 for capsulotomy compared with full control mice of the same strain. ^†^*p* < 0.05, ^††^*p* < 0.01 and ^†††^*p* < 0.001 for WT vs *Ins2*^*Akita*^ capsulotomy groups. ^‡^*p* < 0.05, ^‡‡^*p* < 0.01 and ^‡‡‡^*p* < 0.001 for corneal incision compared with full control mice of the same strain. One-way ANOVA in (**b**) and two-way ANOVA in (**e**–**m**)

A schematic representation of the capsulotomy procedure is shown in electronic supplementary material (ESM) Fig. 1. Briefly, a 1 mm incision was performed in the peripheral cornea and 1% sodium hyaluronate (wt/vol.; Bohus-BioTech, Strömstad, Sweden) administered into the anterior chamber. Capsulotomy was performed in the right eye by cutting the anterior lens capsule using standard micro-scissors. The corneal wound was sutured and topical 2.5% phenylephrine (wt/vol.) /1% atropine administered (wt/vol.). Control mice received corneal incision without capsulotomy in the right eye. Fundus imaging following capsulotomy confirmed the absence of retinal detachment, which may result from corneal puncture [26] (ESM Fig. 1c). Animals showing signs of eye atrophy or anterior chamber/vitreous haemorrhage were excluded from the study. The capsulotomy healed within 1 week of surgery and none of the animals developed lens opacification in the long term (although transient cloudiness was observed for up to 10–15 days). Age-matched mice not receiving capsulotomy or corneal incision served as full controls. Wild-type (WT), *Ins2*^*Akita*^ and *Cx3cr1*^+/GFP^ mice were randomly assigned (using a simple randomisation method) to each surgery group. No specific blinding was carried out to outcome assessment.

### Intraocular administration of anti-LRP6 co-receptor antibody

Blockade of the canonical Wnt signalling pathway was performed via administration of mouse monoclonal antibody against the Wnt low-density lipoprotein receptor-related protein 6 (LRP6) co-receptor (Mab2F1) [27]. Mab2F1 has shown to efficiently reduce the bio-activity of canonical Wnt signalling in rodent eyes at a dose of 20 μg/eye [27]. The surface of mouse retina (5.6 mm^2^) is approximately one-quarter that of rat retina (52 mm^2^) [28]. Therefore, we injected 5 μg/μl MabF1 into mouse eyes. To avoid additional insults from intravitreal injection, 1 μl Mab2F1 (5 μg/μl) or endotoxin-free mouse IgG (5 μg/μl) (MyBioSource, San Diego, CA, USA) in 0.01 mol/l PBS was injected into the anterior chamber immediately after capsulotomy, using a 33 gauge needle (Hamilton-Bonaduz, Bonaduz, Switzerland) cannulated through the sutured wound. Twenty days after capsulotomy, 1 μl Mab2F1 or mouse IgG was administered into the vitreous chamber as previously described [29]. Mab2F1 or IgG treatments were conducted in two separate experiments (*n* ≥ 5 mice per study/group). Age-matched mice not receiving any intravitreal injections served as full controls (*n* ≥ 5 mice per strain).

### Clinical investigations

Fundus images were obtained as previously described [29]. For spectral domain optical coherence tomography (SD-OCT) (*n* = 7 mice for WT full controls; *n* = 5 mice for remaining WT and *Ins2*^*Akita*^ groups), animals were anaesthetised with ketamine/xylazine (90 mg 10 mg^−1^ kg body weight^−1^) and examinations conducted 40 days following capsulotomy (to ensure a prudent duration for the development of retinal degeneration) using the Spectralis Heidelberg OCT system (Heidelberg Engineering, Heidelberg, Germany). Retinal thickness (from nerve fibre layer [NFL] to the photoreceptor outer segments [POS]) was measured at 600 μm eccentricities from the optic disc in dorso–ventral and nasal–temporal sectors.

### Electroretinography

Electroretinography (ERG) responses were recorded 40 days following eye surgery (*n* = 7 mice for WT and *Ins2*^*Akita*^ full controls; *n* = 5 mice for remaining WT and *Ins2*^*Akita*^ groups) as previously described [30, 31]. Scotopic ERGs were taken using mouse corneal ERG electrodes, in response to single white light flashes of different intensities, delivered by a standard Ganzfeld Stimulator (LKC Technologies, Gaithersburg, MD, USA). The amplitudes and implicit times of the scotopic a- and b-waves were obtained.

### qPCR

Total RNA was isolated from mouse retinas 14 days after surgery (*n* = 8 eyes for WT full control; *n* = 6 *Ins2*^*Akita*^ capsulotomy; *n* = 5 for WT and *Ins2*^*Akita*^ remaining groups) using the RNeasy Mini Kit (Qiagen, Crawley, UK). Quantitative PCR (qPCR) was performed using SYBR Green Master (Roche Diagnostics, Mannheim, Germany) in a LightCycler 480 system (Roche Diagnostics). The relative expression levels of target genes (*Vegfa*, *iNos* [also known as *Nos2*], *Il-1β* and *Ccl2*) were normalised to β-actin. The primer sequences used in this study are listed in ESM Table 1.

### Immunoblotting

Ten days after surgery (*n* = 3 eyes/group), retinas were harvested and lysed in RIPA buffer, with protease inhibitor and phosphatase inhibitor cocktails (Sigma-Aldrich, Gillingham, UK). Eluted proteins samples (20 μg) were run on 10% (wt/vol.) SDS-PAGE gels. Samples were probed using rabbit anti-β-catenin (1:2000; Cell Signaling Technology, Danvers, MA, USA) and protein levels compared with β-actin (1:10,000, Santa Cruz Biotechnology, Dallas, TX, USA). Antibodies were validated by the manufacturers for western blot analysis (ESM Table 2).

### Immunohistochemistry and histology

Enucleated globes were dissected and fixed in 2% (wt/vol.) paraformaldehyde and processed for immunohistochemistry as previously described [32]. Retinal sections were stained with primary antibodies (validated by the manufacturers for immunofluorescence analyses [ESM Table 2]) and examined by confocal microscopy (C1-Nikon-Eclipse TE200-U, Nikon UK, Kingston upon Thames, UK). Some retinal sections were processed for H&E staining and examined by light microscopy (Nikon-Eclipse E400 light microscope; Nikon UK).

### Confocal morphometry

Confocal images acquired under constant photomultiplier settings were used for morphometric analysis of retinal neurons, immune cell subsets, Wnt/β-catenin activation, glial fibrillary acidic protein (GFAP)-reactive gliosis and albumin leakage. Images (*n* = 3 mice/group; 2 retinal sections/eye; 4 images/section) were analysed using FIJI software (National Institutes of Health, Bethesda, MD, USA). Morphometric analysis of retinal neurons was conducted as previously described [29, 30]. For a detailed procedure of each morphometric analysis, please refer to the ESM Methods.

### Statistics

ERG scotopic responses were analysed using two-way ANOVA followed by Bonferroni’s post-hoc analysis. SD-OCT retinal thickness and morphometric data in each mouse strain were analysed by one-way ANOVA, followed by Bonferroni’s post hoc analysis using GraphPad Software (La Jolla, CA, USA). Significant outliers in qPCR analysis were identified and discarded using Grubbs’ test (*α* = 0.05, GraphPad). No outliers were detected in the neuronal and immune cells morphometric analysis. Data were expressed as mean ± SEM and *p* < 0.05 was considered statistically significant.

## Results

### Retinal degeneration and scotopic electroretinogram responses following capsulotomy

SD-OCT analysis revealed no major retinal morphological abnormalities in WT and *Ins2*^*Akita*^ mice at 40 days following corneal incision (data not shown) or capsulotomy (Fig. 1b). However, the overall retinal thickness (from the NFL to POS) was significantly reduced in *Ins2*^*Akita*^ corneal incision and capsulotomy groups compared with *Ins2*^*Akit*a^ full controls and with their WT counterparts (Fig. 1b).

Scotopic ERG showed a significant decrease in a-wave amplitudes at higher flash intensities (1, 1.5 log_10_ cd × s/m^2^) in WT mouse eyes 40 days following corneal incision or capsulotomy compared with full control WT eyes (Fig. 1c, e). No significant differences were observed in the amplitude of b-waves (Fig. 1g) or implicit times of a- and b-waves (Fig. 1i, k). In *Ins2*^*Akita*^ mice, a significant reduction in a-wave (Fig. 1f) and b-wave amplitudes (Fig. 1h) was observed in corneal incision (1, 1.5 log_10_ cd × s/m^2^) and capsulotomy (1, 1.5 log_10_ cd × s/m^2^) eyes 40 days after surgery compared with full control *Ins2*^*Akita*^ eyes. The implicit times of a-waves (Fig. 1j) and b-waves (Fig. 1l) were significantly delayed in capsulotomy but not in corneal incision eyes compared with full control eyes and with WT capsulotomy eyes. Overall, these data suggest a greater impairment of retinal electrophysiology following capsulotomy in diabetic eyes.

### Retinal neurodegeneration is worsened in *Ins2*^*Akita*^ diabetic eyes following capsulotomy

**Fig. 2:**
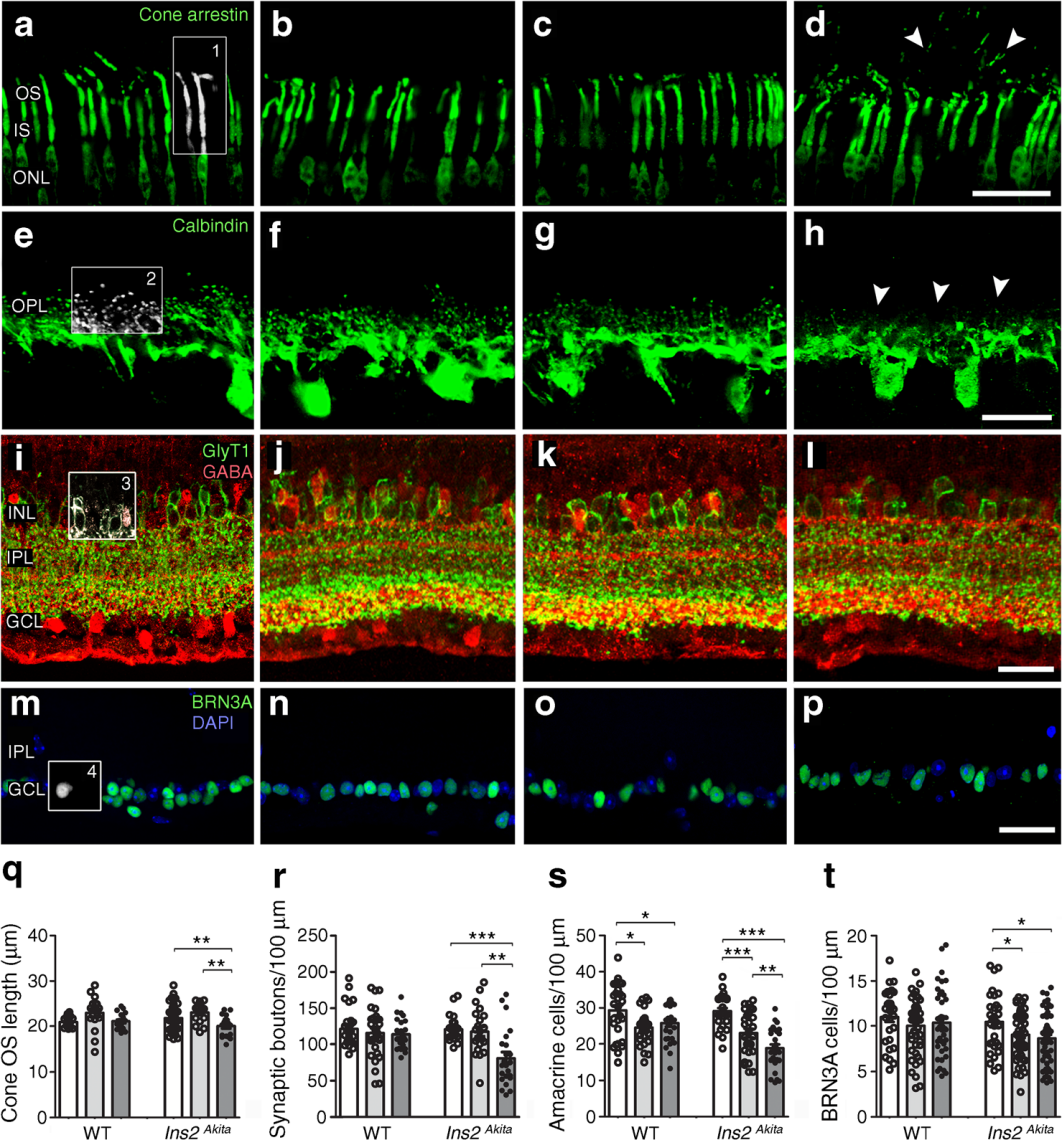
Retinal neurodegeneration in WT and *Ins2*^*Akita*^ mice after capsulotomy. Three-month-old WT (**a**,**b**,**e**,**f**,**i**,**j**,**m**,**n**) and *Ins2*^*Akita*^ (**c**,**d**,**g**,**h**,**k**,**l**,**o**,**p**) mice underwent corneal incision or lens capsulotomy. At 40 days after the procedure, eyes were assessed for neuronal degeneration by immunohistochemistry. (**a**–**p**) Retina photomicrographs of cone photoreceptors (**a**–**d**; cone arrestin), horizontal cells (**e**–**h**; calbindin), amacrine cells (**i**–**l**; red channel, GABA; green channel, glycine transporter 1) or RGCs (**m**–**p**; BRN3A) of WT and *Ins2*^*Akita*^ treatment groups. (**d**) Cone segment disruption (cone arrestin^+^ particles free from cone outer segments [arrowheads]) in *Ins2*^*Akita*^ capsulotomy eyes. (**h**) Loss of horizontal cell synaptic boutons (arrowheads) in *Ins2*^*Akita*^ capsulotomy eyes. (**q**–**t**) Quantitative analysis of (**q**) cone segment length (box 1 in **a**), (**r**) horizontal cell synaptic boutons (box 2 in **e**), (**s**) GABAergic + glycinergic amacrine cells (box 3 in **i**) and (**t**) BRN3A^+^ RGCs (box 4 in **m**) in different treatment groups. White bars, full control; light grey bars, corneal incision; dark grey bars, capsulotomy. *n* = 20 retinal images per strain/condition. Results are presented as mean ± SEM. *p < 0.05, **p < 0.01 and ***p < 0.001 as shown. One-way ANOVA. Scale bars, 20 μm in (**h**) and 30 μm in (**d**, **l**, **p**). GABA, γ-aminobutyric acid; GlyT1, glycine transporter 1; IPL, inner plexiform layer; IS, inner segment; ONL, outer nuclear layer; OS, outer segment

To understand how retinal integrity was affected by capsulotomy, outer and inner neuronal cell populations were characterised 40 days following surgery. A significant reduction in the length of cone photoreceptor segments was observed in *Ins2*^*Akita*^ mice following capsulotomy (cone arrestin; Fig. 2a–d, q), which was accompanied by substantial cone photoreceptor outer segment fragmentation (arrowheads in Fig. 2d). In addition, a significant depletion of horizontal cell dendritic boutons was evident in *Ins2*^*Akita*^ mice receiving capsulotomy (calbindin; Fig. 2e–h, r [see arrowheads in h]).

At the inner retina, the density of amacrine cells (GABAergic + glycinergic) was significantly decreased in both corneal incision and capsulotomy WT groups compared with full control mice (Fig. 2i–j, s). Capsulotomy-induced amacrine cell loss was more severe than corneal-incision-induced amacrine loss in *Ins2*^*Akita*^ mice (Fig. 2k–l, s). The surgery did not affect retinal ganglion cells [RGCs] positive for POU domain, class 4, transcription factor 1 (BRN3A) in WT mice (Fig. 2m–n, t), but significantly reduced the RGC number in *Ins2*^*Akita*^ mice (Fig. 2o–p, t).

### Ocular inflammation is exacerbated in *Ins2*^*Akita*^ diabetic eyes following capsulotomy

**Fig. 3:**
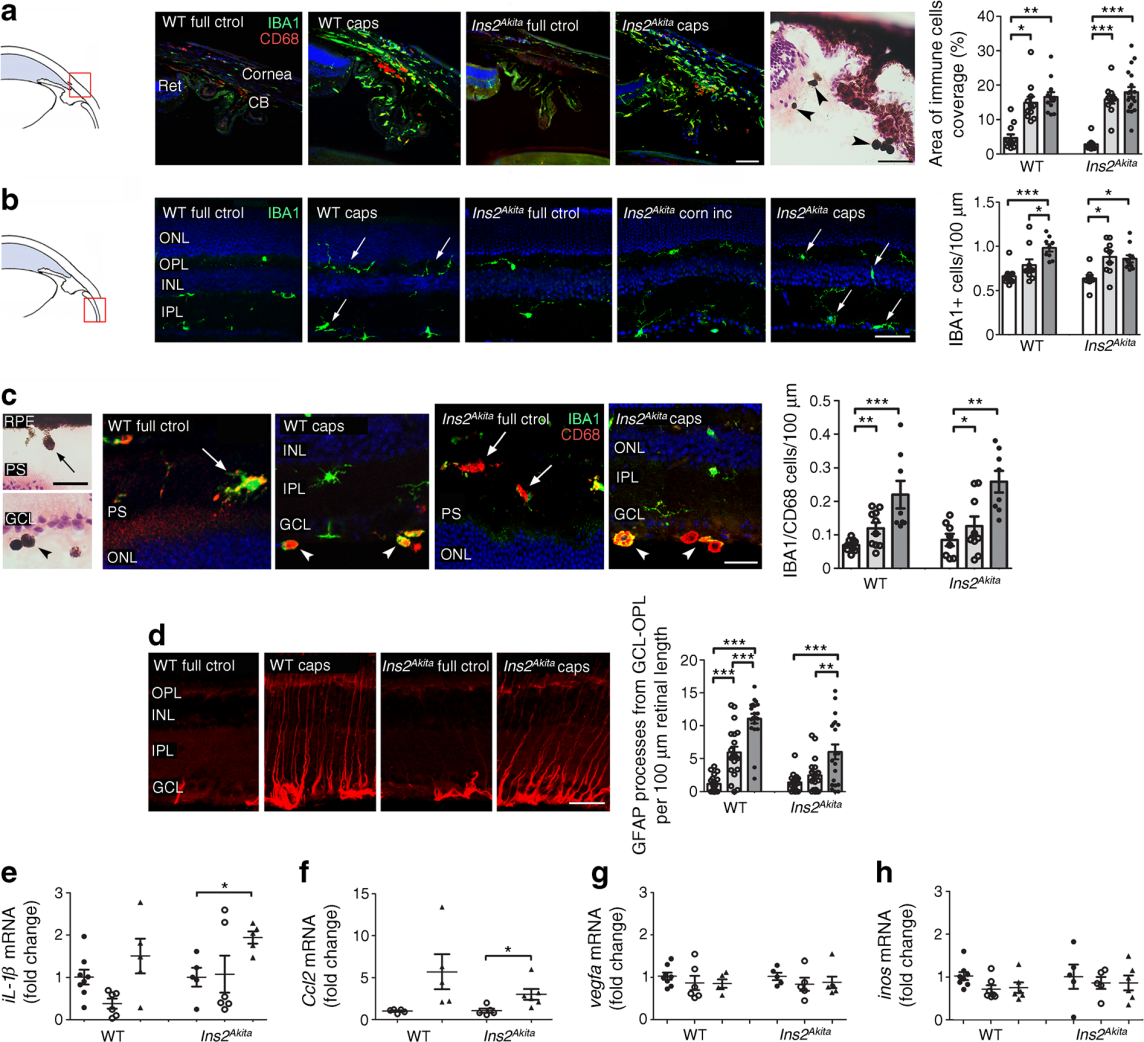
Ocular inflammation in WT and *Ins2*^*Akita*^ mice after capsulotomy. (**a**–**f**) Three-month-old WT and *Ins2*^*Akita*^ mice underwent corneal incision or lens capsulotomy. At 40 days after the procedure, eyes were assessed for inflammation. (**a**) Immunoreactivities in the irido–corneal junction (see diagram) of full control WT and *Ins2*^*Akita*^ eyes or following capsulotomy: red, CD68; green, IBA1. The H&E-stained light micrograph shows infiltrating pigmented cells in the vitreo–retinal border and ciliary body (arrowheads); graph shows the area of IBA1/CD68 cells at the irido–corneal junction in different groups. (**b**) IBA1 immunoreactivity in retina (see diagram) of WT and *Ins2*^*Akita*^ mice of different groups. IBA1^+^ microglial cells were dispersed through all retinal layers following capsulotomy (arrows); graph shows the density of IBA1^+^ cells in different groups. (**c**) H&E-stained light micrographs and CD68 (red) and IBA1 (green) immunoreactivity of WT and *Ins2*^*Akita*^ mouse retinas following capsulotomy. Infiltration of CD68^+^ and IBA1^+^ immune cells in the subretinal space (arrows) and in the vitro–retinal border (arrowheads). The graph shows that a significant increase in IBA1/CD68 cells was observed at the subretinal space and vitro–retinal border of WT and *Ins2*^*Akita*^ capsulotomy eyes. (**d**) GFAP immunoreactivity in middle eccentricities of WT and *Ins2*^*Akita*^ mouse retinas and the density of GFAP^+^ processes expanding from the GCL to the OPL (GCL-OPL) in different treatment groups. (**e**–**h**) qPCR analysis of proinflammatory genes in the retina of full control WT or *Ins2*^*Akita*^ mice 14 days after corneal incision or capsulotomy. In (**a**–**d**): white bars, full control; light grey bars, corneal incision; dark grey bars, capsulotomy. In (**e**–**h**): black circles, full control; white circles, corneal incision; triangles, capsulotomy. In (**a**, **d**), *n* = 12 retinal images per strain/condition. In (**b**, **c**), *n* = 9 retinal sections per strain/condition. In (**e**–**h**), *n* = 8 eyes for WT full control; *n* = 6 eyes for *Ins2*^*Akita*^ capsulotomy; *n* = 5 eyes for WT and *Ins2*^*Akita*^ remaining groups. Results are presented as mean ± SEM. *p < 0.05, **p < 0.01 and ***p < 0.001 as shown. One-way ANOVA. Scale bars, 20 μm in (**c**) both light and fluorescence micrographs; 50 μm in (**a**, **b**, **d**). Caps, capsulotomy; CB, ciliary body; corn inc, corneal incision; ctrol, control; IPL, inner plexiform layer; ONL, outer nuclear layer; PS, photoreceptor segments; Ret, retina; RPE, retinal pigment epithelium

Immunohistochemistry revealed a significant increase in the number of ionised calcium-binding adapter molecule 1 (IBA1)^+^ CD68^+^ cells in the irido–corneal junction of WT and *Ins2*^*Akita*^ mice 40 days following surgery (sham and capsulotomy) (Fig. 3a). H&E staining revealed giant pigmented cells at different ocular locations, including the ciliary body region (arrowheads, Fig. 3a) which also extended to the vitreo–retinal interface (arrowheads in Fig. 3c) and subretinal region (arrows in Fig. 3c).

In the retina, a time-dependent accumulation of IBA1^+^ cells was observed in WT and *Ins2*^*Akita*^ mice following capsulotomy (arrows in ESM Fig. 2). The IBA1^+^ microglia were observed throughout all retinal layers from the NFL to outer plexiform layer (OPL), and the number was significantly higher in the WT and *Ins2*^*Akita*^ mice that underwent capsulotomy compared with full control eyes (arrows in Fig. 3b). In addition, a significantly higher number of IBA1^+^ CD68^+^ cells at the subretinal space (arrows in Fig. 3c) and vitreous–retinal border (arrowheads in Fig. 3c) was observed in WT and *Ins2*^*Akita*^ mice 40 days following capsulotomy compared with corneal incision and full control mice (Fig. 3c). The above increase in immune retinal cell infiltration in capsulotomy eyes was concomitant with exacerbated reactive gliosis (GFAP; Fig. 3d) from the ganglion cell layer (GCL) to the OPL (GCL-OPL) of WT and *Ins2*^*Akita*^ mice (compared with full control and corneal incision groups; Fig. 3d).

To understand whether retinal inflammation precedes capsulotomy-induced retinal degeneration, we examined inflammatory gene expression by qPCR by 14 days post surgery. This analysis revealed a significant upregulation of *Il-1β* mRNA in the retina of *Ins2*^*Akita*^ mice 14 days after capsulotomy compared with non-surgery controls (Fig. 3e). *Ccl2* mRNA levels were below the level of detection in full control WT and *Ins2*^*Akita*^ retinas, becoming detectable following surgery.

Capsulotomy significantly increased *Ccl2* mRNA expression in *Ins2*^*Akita*^ (*p* < 0.05, Fig. 3f) but not WT (*p* = 0.054) mice. No significant changes in the mRNA levels of *Vegfa* and *iNos* were detected in surgery eyes (Fig. 3g, h). Immunohistochemistry staining revealed increased albumin in retinal parenchyma in mice at 10 days post surgery but not 40 days, suggesting transient blood–retinal barrier breakdown (ESM Fig. 3).

### The canonical Wnt signalling pathway is activated in eyes of *Ins2*^*Akita*^ diabetic mice following capsulotomy

**Fig. 4:**
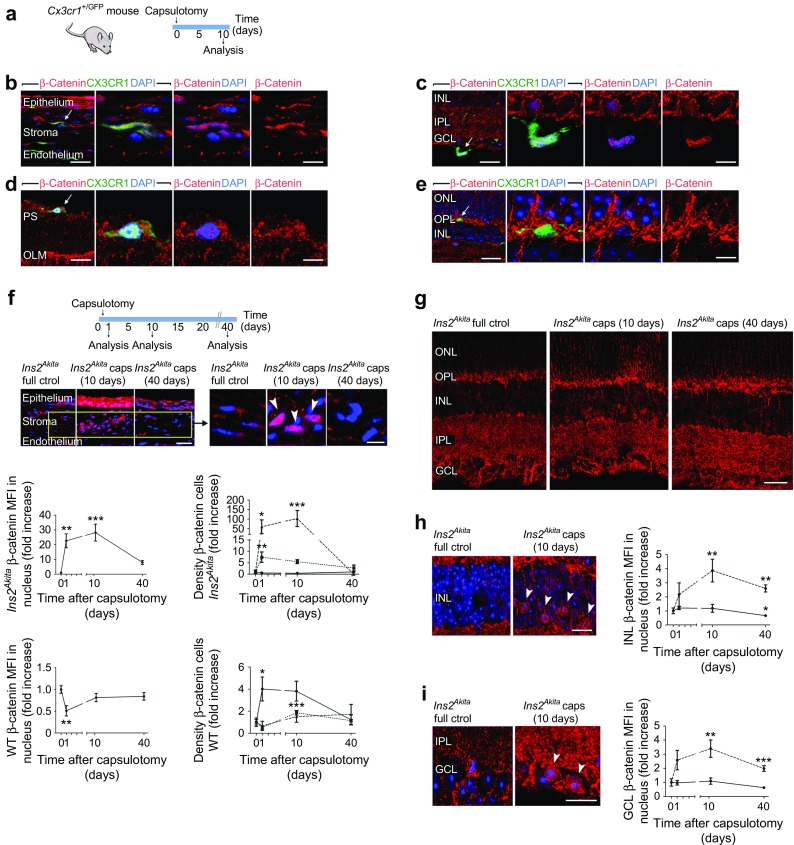
Activation of the canonical Wnt/β-catenin signalling pathway in ocular tissues following capsulotomy. (**a**) Lens capsulotomy was performed in 3-month-old *Cx3cr1*^+/GFP^ mice. After 10 days, eyes were processed for β-catenin immunostaining. (**b**–**e**) GFP^+^ monocyte-derived cells showed nuclear accumulation of β-catenin in stromal cells at the irido–corneal junction (arrow in **b**, with high magnification images to the right), and in cells infiltrating the vitreo–retinal interface (GCL; arrow in **c**, with high magnification images to the right) and subretinal space (PS; arrow in **d**, with high magnification images to the right), but not in retinal microglial cells (arrow in **e**, with high magnification images to the right). (**f**–**i**) Lens capsulotomy was performed in 3-month-old WT and *Ins2*^*Akita*^ mice and eyes collected at different time points (1, 10 and 40 days) after capsulotomy and processed for β-catenin immunostaining. (**f**) Accumulation of β-catenin in the nucleus of stromal cells at the irido–corneal junction. The confocal micrographs show accumulation of β-catenin peaked by 10 days post surgery (arrowheads in high-magnification inset). Graphs show the MFI of β-catenin in the nucleus of stromal cells and the densities of cells accumulating low (solid line), mid (dotted line) and high (dashed line) levels of nuclear β-catenin at different post-surgery times in WT and *Ins2*^*Akita*^ mice. (**g**) Retina micrographs of β-catenin immunoreactivity in *Ins2*^*Akita*^ in different treatment groups. (**h**, **i**) The MFI of β-catenin in the nucleus of INL (**h**, arrowheads) and GCL (**i**, arrowheads), at different post-surgery times in different treatment groups. In (**h**, **i**): solid line, WT; dashed line, *Ins2*^*Akita*^. *n* = 12 (sclero–corneal, **f**) and *n* = 20 (retina, **h**, **i**) images per strain, condition and time point. Results are presented as mean ± SEM. *p < 0.05, **p < 0.01 and ***p < 0.001 compared with full control mice of same strain (as fold change). One-way ANOVA. Scale bars, 30 μm in (**b**–**g**), 20 μm in (**h**, **i**) and 10 μm in the high-magnification insets in (**b**–**f**). Ctrol, control; IPL, inner plexiform layer; ONL, outer nuclear layer; PS, photoreceptor segments; OLM, outer limiting membrane

Activation of the canonical Wnt/β-catenin signalling pathway is known to be involved in various inflammatory responses, including retinal inflammation in diabetic retinopathy [33]. We therefore investigated whether this pathway is activated in immune cells following capsulotomy. Resembling those giant pigmented cells at the retina (Fig. 3c), capsulotomy in *Cx3cr1*^+/GFP^ mice revealed the accumulation of nuclear β-catenin in GFP^+^ monocyte-derived cells at the irido–corneal junction (Fig. 4b), vitreo–retinal border (Fig. 4c) and subretinal space (arrow in Fig. 4d). Interestingly, resident GFP^+^ microglial cells showed negligible levels of nuclear β-catenin following capsulotomy (arrow in Fig. 4e).

We next investigated the dynamics of Wnt/β-catenin activation in ocular tissues following capsulotomy. For this purpose, a detailed morphometric analysis was performed to obtain: (1) the average levels of nuclear β-catenin (measured by mean fluorescence intensity [MFI]; ESM Fig. 4a); and (2) the densities of cells accumulating low, mid and high levels of nuclear β-catenin (ESM Fig. 4b). At the irido–corneal junction, *Ins2*^*Akita*^ mice showed a significant increase in nuclear β-catenin levels in stromal cell populations by 1 day (Fig. 4f, *p* < 0.01) and 10 days (Fig. 4f, *p* < 0.01 [arrowheads show increase in area]) post surgery, which declined to baseline levels by 40 days post surgery. This was accompanied by a transient increase in the densities of cells accumulating mid levels (1 day, *p* < 0.01) and high levels (1 day, *p* < 0.05; 10 days, *p* < 0.001) of nuclear β-catenin (Fig. 4f). WT mice showed no significant increase in the average nuclear β-catenin values of stromal cells (Fig. 4f). However, a transient increase in the densities of cells accumulating low (1 day post surgery, *p* < 0.05) and mid (10 day post surgery, *p* < 0.01) levels of nuclear β-catenin was observed (Fig. 4f).

In the retina, no significant increases in the overall levels of β-catenin were detected by western blot following capsulotomy in WT or *Ins2*^*Akita*^ mice (ESM Fig. 5). However, the immunohistochemical morphometric analysis revealed a rapid and significant upregulation of nuclear β-catenin levels at the GCL (arrowheads in Fig. 4i) and inner nuclear layer (INL) (arrowheads in Fig. 4h) of *Ins2*^*Akita*^ mice. These levels peaked by 10 days post surgery in both the INL (Fig. 4h, *p* < 0.01) and GCL (Fig. 4i, *p* < 0.01), and remained at high levels by 40 days. No significant upregulation of nuclear β-catenin was observed at the GCL or INL of WT mice (Fig. 4h, i). Overall, these data suggest that the canonical Wnt signalling pathway is activated in diabetic eyes following capsulotomy.

### Neutralisation of the canonical Wnt signalling pathway blunts capsulotomy-induced ocular inflammation

**Fig. 5:**
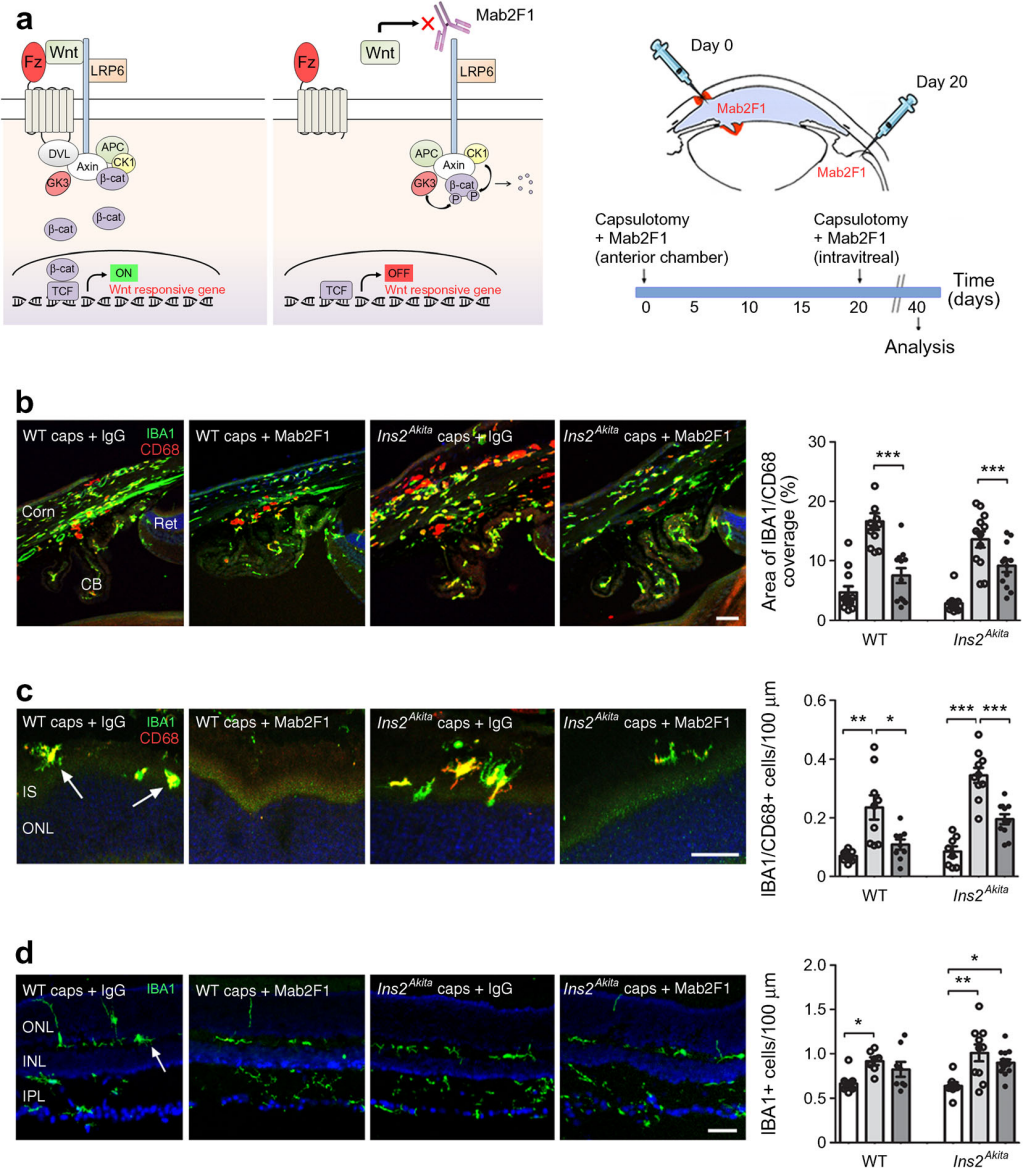
Neutralisation of canonical Wnt/β-catenin signalling pathway by LRP6 antibody suppresses capsulotomy-induced ocular inflammation. (**a**) Wnt/β-catenin signalling pathway was neutralised by administration of monoclonal antibody (Mab2F1) against the LRP6 co-receptor. Mab2F1 or mouse IgG isotype control was administered in 3-month-old WT and *Ins2*^*Akita*^ mice following capsulotomy at day 0 (anterior chamber) and at day 20 (vitreal chamber), and eyes were processed at day 40 for immunohistochemical analysis. (**b**) The area of IBA1 and CD68 at the irido–corneal junction in different treatment groups. (**c**) The density of IBA1 and CD68 in cells at the subretinal space/vitreo–retinal border in different treatment groups. Arrows indicate macrophage infiltration at the subretinal space. (**d**) The density of IBA1^+^ retinal microglial cells in different treatment groups. White bars, full control; light grey bars, capsulotomy + IgG; dark grey bars, capsulotomy + Mab2F1. *n* = 12 sclero–corneal images and *n* = 9 retinal sections per strain and condition. Results are presented as mean ± SEM. *p < 0.05, **p < 0.01 and ***p < 0.001 as shown. One-way ANOVA. Scale bars, 20 μm. APC, adenomatous polyposis coli protein; β-cat, β-catenin; caps, capsulotomy; CB, ciliary body; CK1, casein kinase 1α; Corn, cornea; DVL, Dishevelled; GK3, glycogen synthase kinase 3; IPL, inner plexiform layer; IS, inner segment; ONL, outer nuclear layer; ret, retina; TCF, T-cell factor

We then investigated whether inhibiting canonical Wnt signalling could reduce capsulotomy-induced inflammation. WT and *Ins2*^*Akita*^ capsulotomy eyes were treated with monoclonal antibody against the Wnt LRP6 co-receptor (Mab2F1) [27] or mouse IgG isotype control (Fig. 5a). Mab2F1 treatment significantly reduced the density of IBA1^+^ CD68^+^ infiltrating cells at the irido–corneal junction, subretinal space and vitreo–retinal border in both WT and *Ins2*^*Akita*^ mice 40 days post surgery (Fig. 5b, c). Mab2F1 did not affect the density of retinal microglial cells (Fig. 5d). This was consistent with the nuclear levels of β-catenin in immune cells observed in *Cx3cr1*^+/GFP^ mice following capsulotomy (Fig. 4e). Furthermore, Mab2F1 downregulated surgery-induced upregulation of the nuclear β-catenin levels in the GCL and INL in *Ins2*^*Akita*^ mice (*p* < 0.01; ESM Fig. 6).

### Mab2F1 treatment ameliorates capsulotomy-induced retinal neurodegeneration

**Fig. 6:**
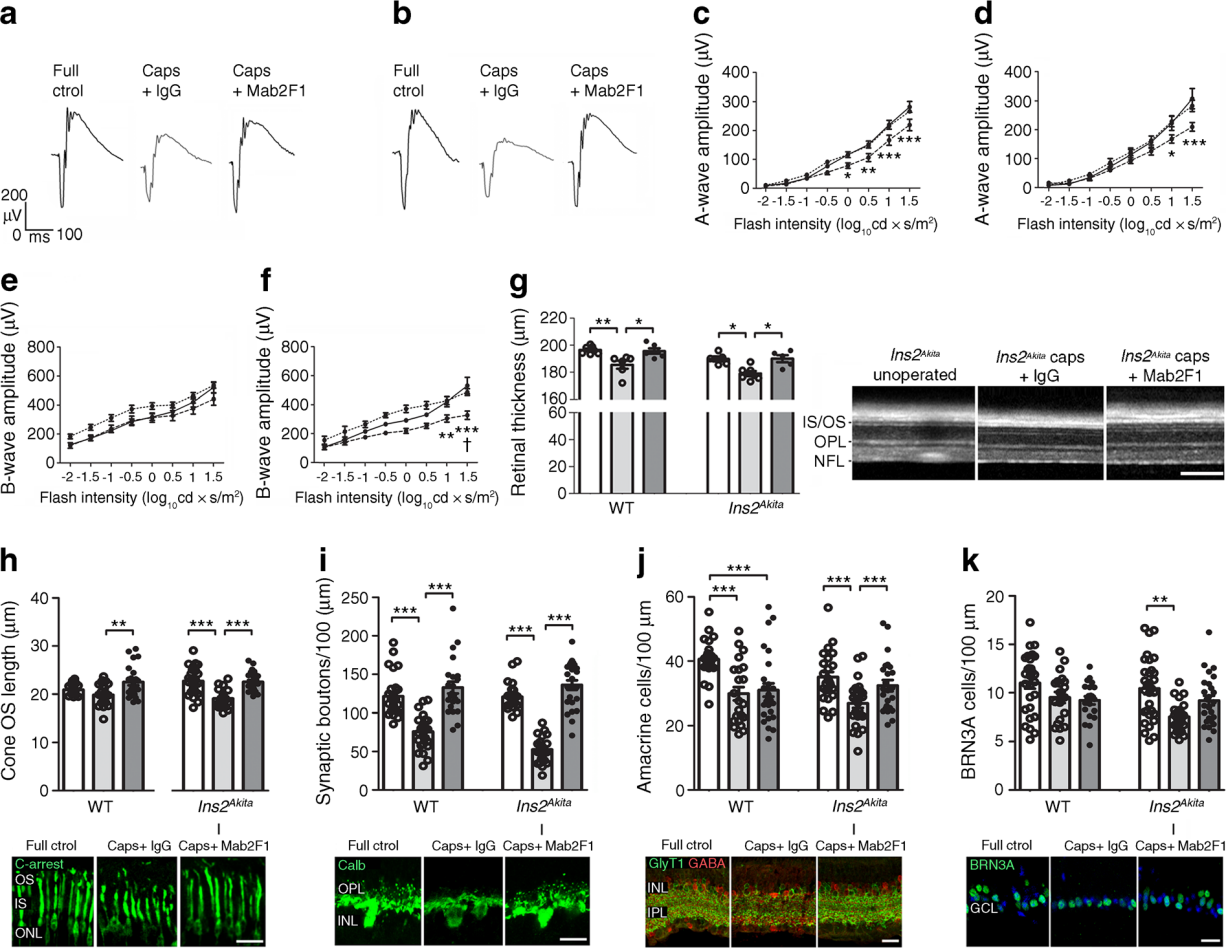
The effect of Mab2F1 in capsulotomy-induced retinal degeneration. Mab2F1 or mouse IgG isotype control was administered in 3-month-old WT and *Ins2*^*Akita*^ mice following capsulotomy at day 0 (anterior chamber) and at day 20 (intravitreal). At 40 days after the procedure, eyes were assessed for retinal function (ERG) and retinal degeneration (SD-OCT and analysis of neuronal morphometry). (**a**, **b**) Representative scotopic ERG responses from WT (**a**) and *Ins2*^*Akita*^ (**b**) mice. (**c**–**f**) The amplitude (μV) of scotopic a-wave (**c**, **d**) and b-wave (**e**, **f**) in WT (**c**, **e**) and *Ins2*^*Akita*^ (**d**, **f**) mice of different treatment groups. (**g**) Quantitative analysis of neuroretinal thickness by SD-OCT in WT and *Ins2*^*Akita*^ mice in different treatment groups, with representative SD-OCT images among the different *Ins2*^*Akita*^ treatment groups. (**h**) The length of cone photoreceptor segments (cone arrestin) and the densities of (**i**) horizontal cell synaptic boutons (calbindin), (**j**) amacrine cells (GABAergic + glycinergic) and (**k**) retinal ganglion cells (BRN3A) in WT and *Ins2*^*Akita*^ mice of different treatment groups, with representative confocal micrographs of the different *Ins2*^*Akita*^ treatment groups shown below each graph. In (**c**–**f**): solid line, full control; dashed line, capsulotomy + IgG; dotted line, capsulotomy + Mab2F1. In (**g** and **h**–**k**): white bars, full control; light grey bars, capsulotomy + IgG; dark grey bars, capsulotomy + Mab2F1. For ERG and SD-OCT (**c**–**g**), *n* = 7 for WT full controls and *n* = 5 for WT and *Ins2*^*Akita*^ remaining groups. For neuronal morphometry (**h**–**k**), *n* = 24 retinal images per strain and condition. ERG white light flashes are presented as log10 cd × s/m^2^. Results are presented as mean ± SEM. (**c**–**f**) *p < 0.05, **p < 0.01 and ***p < 0.001 compared with full control or IgG-treated mice of same strain. (**e**–**f**) ^†^*p* < 0.05 between WT and *Ins2*^*Akita*^ IgG groups. (**h**–**k**) **p < 0.01 and ***p < 0.001 as shown. Two-way ANOVA used in (**c**–**f**) and one-way ANOVA in (**g**–**k**). Scale bars, 200 μm in (**g**) and 20 μm in (**h**–**k**). Calb, calbindin; c-arrest, cone arrestin; caps, capsulotomy; ctrol, control; GlyT1, glycine transporter 1; IPL, inner plexiform layer; IS, inner segment; ONL, outer nuclear layer; OS, outer segment

Treatment with Mab2F1 significantly prevented the reduction of a- and b-wave scotopic ERG responses (Fig. 6a–f) and the capsulotomy-induced retinal thinning (SD-OCT, Fig. 6g). Immunohistochemistry showed that treatment with Mab2F1 significantly prevented the capsulotomy-induced shortening of cone outer segments in *Ins2*^*Akita*^ mice (cone arrestin; Fig. 6h). Synaptic boutons at the OPL were preserved by Mab2F1 treatment in both WT and *Ins2*^*Akita*^ mice (calbindin; Fig. 6i). In addition, the treatment preserved amacrine cells (GABAergic + glycinergic; Fig. 6j) and RGCs (BRN3A; Fig. 6k) in *Ins2*^*Akita*^ mice. Overall, neutralisation of the canonical Wnt signalling pathway by monoclonal antibody resulted in the prevention of capsulotomy-induced retinal neurodegeneration in the diabetic and non-diabetic context.

## Discussion

In this study, we show that lens capsulotomy triggers inflammation in the anterior and posterior eye segments, which is associated with retinal neurodegeneration. We also show that capsulotomy-induced ocular inflammation and retinal neurodegeneration is worsened in the diabetic context, as observed in *Ins2*^*Akita*^ mice. Furthermore, our data suggest capsulotomy-induced retinal neurodegeneration is driven by ocular inflammation through Wnt/β-catenin signalling. Consequently, blocking this pathway alleviated capsulotomy-induced retinal neurodegeneration in WT and *Ins2*^*Akita*^ mice.

Although the immune cell phenotype remains elusive, lens capsulotomy promoted the infiltration of IBA1^+^ CD68^+^ cells at the irido–corneal junction. Clinically, cataract-surgery-induced inflammation is characterised by flare and immune cell infiltration of the anterior chamber and the presence of pro-inflammatory chemokines, including IL-1β in the aqueous humour [34, 35]. Cataract-surgery-induced immune response at the posterior eye segment has not been investigated clinically. In this study, we have shown that lens capsulotomy triggered retinal inflammation, characterised by infiltration of IBA1^+^ CD68^+^ cells at the subretinal space and vitreo–retinal border, increase in retinal microglial cells and upregulation of pro-inflammatory gene expression. Previously, we reported the upregulation of inflammatory mediators such as chemokine [C-C motif] ligand 2 (CCL2), IL-1β and complement proteins following extracapsular lens extraction in mice [19]. In this study, lens capsulotomy marginally increased *Ccl2* but not *Il-1β* expression in WT mouse retina. This discrepancy may reflect the minimal surgical insult of capsulotomy surgery compared with extracapsular lens extraction, as ocular inflammation with cataract surgery is related to surgical trauma [18]. Unsurprisingly, capsulotomy-induced retinal inflammation was further exacerbated in *Ins2*^*Akita*^ mice, featured by *IL-1β* upregulation. The upregulation of this chemokine is crucial, given its critical role in inflammation [36]. A chronic low-grade inflammation, including upregulation of cytokines and chemokines, is known to exist in the diabetic retina [3, 5]. Such a pre-existing condition may therefore exacerbate capsulotomy-mediated retinal inflammation.

Capsulotomy in 3-month-old *Ins2*^*Akita*^ diabetic mice triggered early onset of diabetic retinopathy-related neurodegeneration, including the disruption of synaptic structures at the OPL and loss of amacrine cells and RGCs [30]. This may be related to increased inflammation (e.g. higher *Il-1β* expression) in diabetic eyes as inflammatory mediators are known to have detrimental effects on retinal neurons. Infiltrating immune cells may play a critical role in this process through the secretion of various proinflammatory cytokines and growth factors [20, 37]. Accordingly, retinal damage was significantly alleviated in WT and *Ins2*^*Akita*^ Mab2F1-treated eyes, which displayed reduced immune cell infiltration in the anterior and posterior eye segments following capsulotomy.

Activation of Wnt/β-catenin signalling is shown to exert a detrimental effect in capsulotomy-induced retinal damage. Consequently, capsulotomy-induced retinal damage in WT and *Ins2*^*Akita*^ mice was prevented by Mab2F1 treatment. In diabetic retinopathy, previous investigations have suggested that Wnt/β-catenin signalling may play an immunopathogenic role [21, 38]. Accordingly, upregulation of nuclear β-catenin was exacerbated in the anterior and posterior eye segments of *Ins2*^*Akita*^ mice following lens capsulotomy. The precise role of such upregulation is, however, uncertain. For example, at the inflammatory level, activation of Wnt/β-catenin signalling is known to exert proinflammatory functions [23, 39] and to be activated in macrophages during the early stages of wound repair [40]. Hence, the observation of increased nuclear β-catenin in monocyte-derived cells shortly after capsulotomy at the irido–corneal junction, subretinal space and vitreo–retinal border is not surprising. Activation of Wnt/β-catenin in the aforementioned immune cell subsets may induce inflammation over tissue repair [23, 40], as validated by Mab2F1 treatment. These studies, including ours, suggest a potential therapeutic effect in targeting Wnt/β-catenin signalling in diabetic individuals undergoing cataract surgery.

Previous studies have shown that activation of Wnt/β-catenin in neurons is protective in the central nervous system [41, 42]. The upregulation of nuclear β-catenin in non-immune cells (likely neurons) in the *Ins2*^*Akita*^ retina may thus reflect a compensatory mechanism to favour neuronal survival. In line with this, in unpublished observations (Jose R. Hombrebueno, Heping Xu) repeated intravitreal injection of Mab2F1 failed to alleviate retinal neurodegeneration in full control *Ins2*^*Akita*^ mice, including abnormal scotopic ERG responses, SD-OCT retinal thinning and RGC degeneration. Further, previous studies have shown that activation of Wnt/β-catenin signalling via intravitreal injection with recombinant Wnt3A induced RGC survival in a mouse model of axon crush injury [43] and preservation of photoreceptors in a mouse model of inherited retinal degeneration [44]. Nevertheless, a deleterious role of Wnt/β-catenin signalling in retinal neurons cannot be completely disregarded [45].

Some factors may limit the translation of our study to clinical practice. Phacoemulsification is the most common clinical procedure for cataract surgery, but is impossible to perform in the mouse eye. Extracapsular lens extraction was conducted initially, but it triggered severe inflammation [19] and was always followed by lens opacification [25]. Furthermore, extracapsular lens extraction (without intraocular lens implantation) is rarely performed in individuals with cataracts and diabetes. The procedure was therefore discarded for this study. We opted for small modifications to mimic cataract surgery by phacoemulsification, such as the small surgical wound and minimal disruption of lens structure. Interestingly, we observed that the capsule re-sealed following surgery and the lens maintained transparency after a prudent time (i.e. 20 days). The transient opacification of the cornea and lens (lasting up to 10–15 days) following surgery prevented us from conducting SD-OCT and fundus examinations during that period. The SD-OCT measurement on day 40 after surgery did not show any signs of retina oedema. Although we detected albumin leakage, indicative of blood–retinal barrier breakdown [46], the microscopic levels of leakage may not cause significant retinal oedema. The lack of direct evidence of surgery-induced retinal oedema in diabetic mouse eyes makes it difficult to justify the value of this model to study the mechanism of cataract-surgery-mediated macular oedema in people with diabetes. This is another limitation of the study. Finally, it is possible that the inflammation observed in the anterior chamber could be partially due to the retained lens fragments from ruptured capsule [47].

In summary, this study provides novel insights into cataract-surgery-mediated retinal complications in diabetic and non-diabetic individuals. This is of special importance, given: (1) the absence of established guidelines for the postoperative treatment of inflammation induced by cataract surgery [48]; (2) the limitation and adverse side effects of current cataract surgery postoperative treatments (corticosteroids) [49]; and (3) the contraindication of cataract surgery in diabetes [50]. Despite the limitations of scaling cataract surgery in the small mouse eyes, further study in large animals or clinical trials will determine the suitability of targeting the Wnt/β-catenin signalling pathway for the management of postoperative retinal complication in diabetes.

## Electronic supplementary material

ESM (PDF 817 kb)

## Data Availability

Data are available on request from the authors.

## Abbreviations

BRN3A: POU domain, class 4, transcription factor 1
ERG: Electroretinography
GCL: Ganglion cell layer
GFAP: Glial fibrillary acidic protein
GFP: Green fluorescent protein
IBA1: Ionised calcium-binding adapter molecule 1
INL: Inner nuclear layer
LRP6: Low-density lipoprotein receptor-related protein 6
MFI: Mean fluorescence intensity
NFL: Nerve fibre layer
OPL: Outer plexiform layer
POS: Photoreceptor outer segments
qPCR: Quantitative PCR
RGC: Retinal ganglion cell
SD-OCT: Spectral domain optical coherence tomography
WT: wild-type

## References

[1] Gadsby R (2002). Epidemiology of diabetes. Adv Drug Deliv Rev.

[2] Fong DS, Aiello LP, Ferris FL, Klein R (2004). Diabetic retinopathy. Diabetes Care.

[3] Gardner TW, Antonetti DA, Barber AJ, LaNoue KF, Levison SW (2002). Diabetic retinopathy: more than meets the eye. Surv Ophthalmol.

[4] Stitt AW, Curtis TM, Chen M (2016). The progress in understanding and treatment of diabetic retinopathy. Prog Retin Eye Res.

[5] Tang J, Kern TS (2011). Inflammation in diabetic retinopathy. Prog Retin Eye Res.

[6] Jeganathan VS, Wang JJ, Wong TY (2008). Ocular associations of diabetes other than diabetic retinopathy. Diabetes Care.

[7] Bourne RRA, Stevens GA, White RA (2013). Causes of vision loss worldwide, 1990-2010: a systematic analysis. Lancet Glob Health.

[8] Hennis A, Wu SY, Nemesure B, Leske C (2004). Risk factors for incident cortical and posterior subcapsular lens opacities in the Barbados eye studies. Arch Ophthalmol.

[9] Li L, Wan XH, Zhao GH (2014) Meta-analysis of the risk of cataract in type 2 diabetes. BMC Ophthalmol 14:94 10.1186/1471-2415-14-94 PMC4113025 25060855

[10] Haddad NMN, Sun JK, Abujaber S, Schlossman DK, Silva PS (2014). Cataract surgery and its complications in diabetic patients. Semin Ophthalmol.

[11] Chew EY, Benson WE, Remaley NA (1999). Results after lens extraction in patients with diabetic retinopathy—early treatment diabetic retinopathy study report number 25. Arch Ophthalmol.

[12] Antcliff RJ, Poulson A, Flanagan DW (1996). Phacoemulsification in diabetics. Eye.

[13] Henricsson M, Heijl A, Janzon L (1996). Diabetic retinopathy before and after cataract surgery. British Journal of Ophthalmology.

[14] Hong T, Mitchell P, de Loryn T, Rochtchina E, Cugati S, Wang JJ (2009). Development and progression of diabetic retinopathy 12 months after phacoemulsification cataract surgery. Ophthalmology.

[15] Samanta Anupam, Kumar Premranjan, Machhua Sanghamitra, Rao G Nageswar, Pal Arttatrana (2014). Incidence of cystoid macular oedema in diabetic patients after phacoemulsification and free radical link to its pathogenesis. British Journal of Ophthalmology.

[16] Hayashi K, Igarashi C, Hirata A, Hayashi H (2009). Changes in diabetic macular oedema after phacoemulsification surgery. Eye.

[17] Pande MV, Spalton DJ, Kerr-Muir MG, Marshall J (1996). Postoperative inflammatory response to phacoemulsification and extracapsular cataract surgery: aqueous flare and cells. J Cataract Refract Surg.

[18] Oshika T, Yoshimura K, Miyata N (1992). Postsurgical inflammation after phacoemulsification and extracapsular extraction with soft or conventional intraocular lens implantation. J Cataract Refract Surg.

[19] Xu H, Chen M, Forrester JV, Lois N (2011). Cataract surgery induces retinal pro-inflammatory gene expression and protein secretion. Invest Ophthalmol Vis Sci.

[20] Meleth AD, Agron E, Chan CC (2005). Serum inflammatory markers in diabetic retinopathy. Invest Ophthalmol Vis Sci.

[21] Chen Y, Hu Y, Zhou T (2009). Activation of the Wnt pathway plays a pathogenic role in diabetic retinopathy in humans and animal models. Am J Pathol.

[22] Halleskog C, Mulder J, Dahlstrom J (2011). WNT signaling in activated microglia is proinflammatory. Glia.

[23] Ma B, Hottiger MO (2016). Crosstalk between wnt/beta-catenin and NF-kappa B signaling pathway during inflammation. Front Immunol.

[24] Yoshioka M, Kayo T, Ikeda T, Koizumi A (1997). A novel locus, Mody4, distal to D7Mit189 on chromosome 7 determines early-onset NIDDM in nonobese C57BL/6 (Akita) mutant mice. Diabetes.

[25] Lois N, Taylor J, McKinnon AD, Forrester JV (2005). Posterior capsule opacification in mice. Arch Ophthalmol.

[26] Zeng R, Zhang Y, Shi F, Kong F (2012). A novel experimental mouse model of retinal detachment: complete functional and histologic recovery of the retina. Invest Ophthalmol Vis Sci.

[27] Lee K, Hu Y, Ding L (2012). Therapeutic potential of a monoclonal antibody blocking the Wnt pathway in diabetic retinopathy. Diabetes.

[28] Remtulla S, Hallett PE (1985). A schematic eye for the mouse, and comparisons with the rat. Vis Res.

[29] Hombrebueno JR, Luo C, Guo L, Chen M, Xu H (2014). Intravitreal injection of normal saline induces retinal degeneration in the C57BL/6J mouse. Transl Vis Sci Technol.

[30] Hombrebueno JR, Chen M, Penalva RG, Xu H (2014). Loss of synaptic connectivity, particularly in second order neurons is a key feature of diabetic retinal neuropathy in the Ins2Akita mouse. PLoS One.

[31] Hombrebueno JR, Ali IH, Xu H, Chen M (2015). Sustained intraocular VEGF neutralization results in retinal neurodegeneration in the Ins2(Akita) diabetic mouse. Sci Rep.

[32] Hombrebueno JR, Tsai MM, Kim HL, De Juan J, Grzywacz NM, Lee EJ (2010). Morphological changes of short-wavelength cones in the developing S334ter-3 transgenic rat. Brain Res.

[33] Chen Qian, Ma Jian-xing (2017). Canonical Wnt signaling in diabetic retinopathy. Vision Research.

[34] Cho H, Wolf KJ, Wolf EJ (2009). Management of ocular inflammation and pain following cataract surgery: focus on bromfenac ophthalmic solution. Clin Ophthalmol.

[35] Raizman MB, Donnenfeld ED, Weinstein AJ (2007). Clinical comparison of two topical prednisolone acetate 1% formulations in reducing inflammation after cataract surgery. Curr Med Res Opin.

[36] Dinarello CA (2011). A clinical perspective of IL-1β as the gatekeeper of inflammation. Eur J Immunol.

[37] Takagi H, Otani A, Kiryu J, Ogura Y (1999). New surgical approach for removing massive foveal hard exudates in diabetic macular edema. Ophthalmology.

[38] Zhao L, Patel SH, Pei J, Zhang K (2013). Antagonizing Wnt pathway in diabetic retinopathy. Diabetes.

[39] Suryawanshi A, Tadagavadi RK, Swafford D, Manicassamy S (2016). Modulation of inflammatory responses by Wnt/beta-catenin signaling in dendritic cells: a novel immunotherapy target for autoimmunity and cancer. Front Immunol.

[40] Amini-Nik S, Cambridge E, Yu W (2014). Beta-catenin-regulated myeloid cell adhesion and migration determine wound healing. J Clin Invest.

[41] Zhang L, Yan R, Zhang Q (2013). Survivin, a key component of the Wnt/beta-catenin signaling pathway, contributes to traumatic brain injury-induced adult neurogenesis in the mouse dentate gyrus. Int J Mol Med.

[42] Gao K, Shen ZL, Yuan YJ (2016). Simvastatin inhibits neural cell apoptosis and promotes locomotor recovery via activation of Wnt/beta-catenin signaling pathway after spinal cord injury. J Neurochem.

[43] Patel AK, Park KK, Hackam AS (2017). Wnt signaling promotes axonal regeneration following optic nerve injury in the mouse. Neuroscience.

[44] Patel AK, Surapaneni K, Yi H (2015). Activation of Wnt/beta-catenin signaling in Muller glia protects photoreceptors in a mouse model of inherited retinal degeneration. Neuropharmacology.

[45] Libro R, Bramanti P, Mazzon E (2016). The role of the Wnt canonical signaling in neurodegenerative diseases. Life Sci.

[46] Canning P, Kenny BA, Prise V (2016). Lipoprotein-associated phospholipase A(2) (Lp-PLA(2)) as a therapeutic target to prevent retinal vasopermeability during diabetes. Proc Natl Acad Sci U S A.

[47] Van Der Woerdt A (2000). Lens-induced uveitis. Vet Ophthalmol.

[48] Olson RJ, Braga-Mele R, Chen SH (2017). Cataract in the Adult Eye Preferred Practice Pattern(R). Ophthalmology.

[49] McGhee CN, Dean S, Danesh-Meyer H (2002). Locally administered ocular corticosteroids: benefits and risks. Drug Saf.

[50] Pollreisz A, Schmidt-Erfurth U (2010). Diabetic cataract—pathogenesis, epidemiology and treatment. J Ophthalmol.

