# Supplementary material for: Antagonising Wnt/β-catenin signalling ameliorates lens-capsulotomy-induced retinal degeneration in a mouse model of diabetes
Source: Diabetologia. 2018 Jul 17;61(11):2433–46. doi: 10.1007/s00125-018-4682-3 (PMC6182657; doi:10.1007/s00125-018-4682-3)
Supplement: Supplementary file 1 — (PDF 817 kb) [file 125_2018_4682_MOESM1_ESM.pdf]

# Antagonizing Wnt/β-catenin signalling ameliorates lens capsulotomy-induced retinal degeneration in diabetes

Jose R. Hombrebueno[1, *, #], Imran HA. Ali[1*], Jian-xing Ma[2], Mei Chen[1, #] and Heping Xu[1, #]

[1]Centre for Experimental Medicine, School of Medicine, Dentistry and Biomedical Sciences, Queen's University Belfast, Belfast, UK.

[2]Department of Physiology, Harold Hamm Diabetes Center, University of Oklahoma Health Sciences Center, Oklahoma City, OK 73104, United States

*Jose R. Hombrebueno and Imran HA. Ali contributed equally to this work

[#], Corresponding authors: Professor Heping Xu, Centre for Experimental Medicine, the Wellcome-Wolfson Institute of Experimental Medicine, School of Medicine, Dentistry and Biomedical Sciences, Queen's University Belfast, 97 Lisburn Road, Belfast, BT9 7BL, UK.

Tel:+44(0)2890976463

heping.xu@qub.ac.uk, or Dr Mei Chen: m.chen@qub.ac.uk,

or Dr Jose R. Hombrebueno: j.romero@qub.ac.uk

**ESM Methods**

**Confocal morphometry**

Confocal images were used for morphometric analysis of retinal neurons (40-days after eye surgery), albumin leakage (5- and 40- days post-surgery), GFAP reactive gliosis, Iba1$^{+}$CD68$^{+}$ cells at the sclera-corneal junction (40-days post-surgery) and Wnt/β-catenin activation (1-, 5-, 10- and 40-days post-surgery). Images (n≥3 mice/group; 2 retinal sections/eye; 4 images/section) were analysed using FIJI software (National Institutes of Health, Bethesda, USA). For neuronal morphometry, the following parameters were analysed: 1) cone segment length (outer segment plus inner segment), 2) horizontal cell synaptic bouton density, 3) GABAergic and glycinergic amacrine cell density and 4) Retinal ganglion cell density. At least 20 retinal images per strain/treatment were analysed and values averaged and normalized to 100 µm retinal length.

Vascular leakage was assessed by quantifying the mean fluorescence intensity [MFI] of albumin immunostaining from confocal images captured under constant photomultiplier settings, in which the mean luminance values (average brightness per pixel) were calculated from manually traced areas comprising the inner segments of photoreceptors to the ganglion cell layer. Background images were acquired from a vacant area of the labeled section and subtracted from the raw images to eliminate background noise. Reactive gliosis was assessed by quantifying the density of GFAP$^{+}$ processes expanding from the GCL to the OPL. At least 20 retinal images per strain/treatment were analysed and values averaged and normalized to 100 µm retinal length.

The infiltration of immune cells at the sclera-corneal junction was assessed by calculating the percentage of Iba1$^{+}$CD68$^{+}$ cell coverage in corneal tissues (from corneal epithelium to endothelium). At least 12 retinal images per strain/treatment were analysed. Activation of Wnt/β-catenin signalling was obtained as shown in ESM Fig 4, by assessing a) the mean fluorescence intensity [MFI] of β-catenin in cell nuclei and b) the density of low-, mid- and high-β-catenin cells at the irido-corneal junction.

The density of $Iba1^{+}CD68^{+}$ cells at the subretinal space/vitreo-retinal border and the density of $Iba1^{+}$ retinal microglia were quantified *in situ* by epifluorescence microscopy, across whole retinal sections using a Nikon Eclipse E400 light microscope (to allow for the precise quantification of cellular bodies) Cell densities were then normalized to 100 µm/retinal length. At least 9 retinal sections (n≥3 mice/group, 3 retinal sections/eye) were analysed.

**ESM Table 1.** Primers used for real time RT-PCR

| Genes | Forward | Reverse |
|---|---|---|
| *IL-1b* | TCCTTGTGCAAGTGTCTGAAGC | ATGAGTGATACTGCCTGCCTGA |
| *iNOS* | GGCAAACCCAAGGTCTACGTT | TCGCTCAAGTTCAGCTTGGT |
| *VEGFa* | CCCACGTCAGAGAGCAACAT | TTTCTTGCGCTTTCGTTTTT |
| *CCL2* | AGCCAGATGCAGTTAACGC | CTGATCTCATTTGGTTCCGA |
| *β-actin* | AGGGGAGAGCGGGTAAGAGA | GGACAGGACTAGGCGGAACA |

**ESM Table 2.** Primary antibodies used in the study

| Antigen | **Antiserum** (catalogue number) | Dilution | Source |
|---|---|---|---|
| Albumin | goat anti-albumin (A80-129A) | 1:200 | Bethyl |
| β-catenin | rabbit anti-β-catenin (#8480) | 1:200 | Cell Signaling |
| β-actin | mouse anti-β-actin (sc-47778) | 1:10000 | Santa Cruz |
| Brn3a | goat anti-Brn3a (sc-31984) | 1:500 | Santa Cruz |
| Calbindin | rabbit anti-calbindin (AB1778) | 1:1000 | Chemicon |
| CD68 | rat anti-CD68 (MCA1957) | 1:200 | AbD Serotec |
| Cone arrestin | rabbit anti-cone arrestin (AB15282) | 1:10000 | Chemicon |
| γ-aminobutyric acid (GABA) | rabbit anti-GABA (A2052) | 1:500 | Sigma |
| Glial fibrillary acidic protein (GFAP) | rabbit anti-GFAP (ab7260) | 1:200 | Abcam |
| Glycine Transporter-1 (GlyT1) | goat anti-glyT1 (AB1770) | 1:3000 | Chemicon |
| Iba-1 | rabbit anti-Iba1 (019-19741) | 1:400 | Wako |

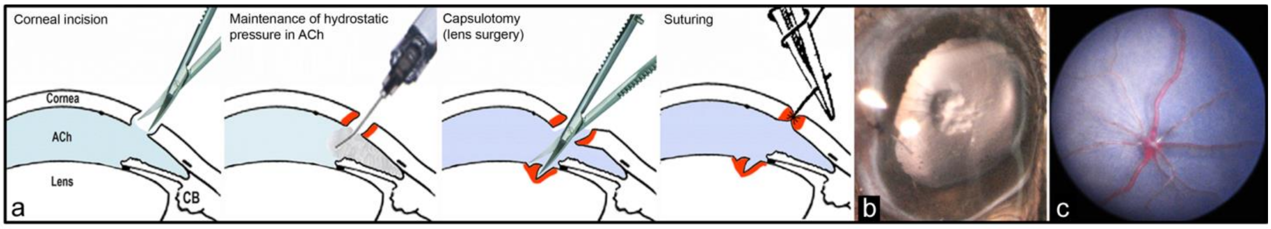


**ESM Figure 1. Lens-Capsulotomy surgery in mouse eyes.** (a-b) Capsulotomy conducted in the mouse eye. (c) Fundus image in *Ins2*$^{Akita}$ capsulotomy eye at day 0. ACh, anterior chamber; CB, ciliary body.

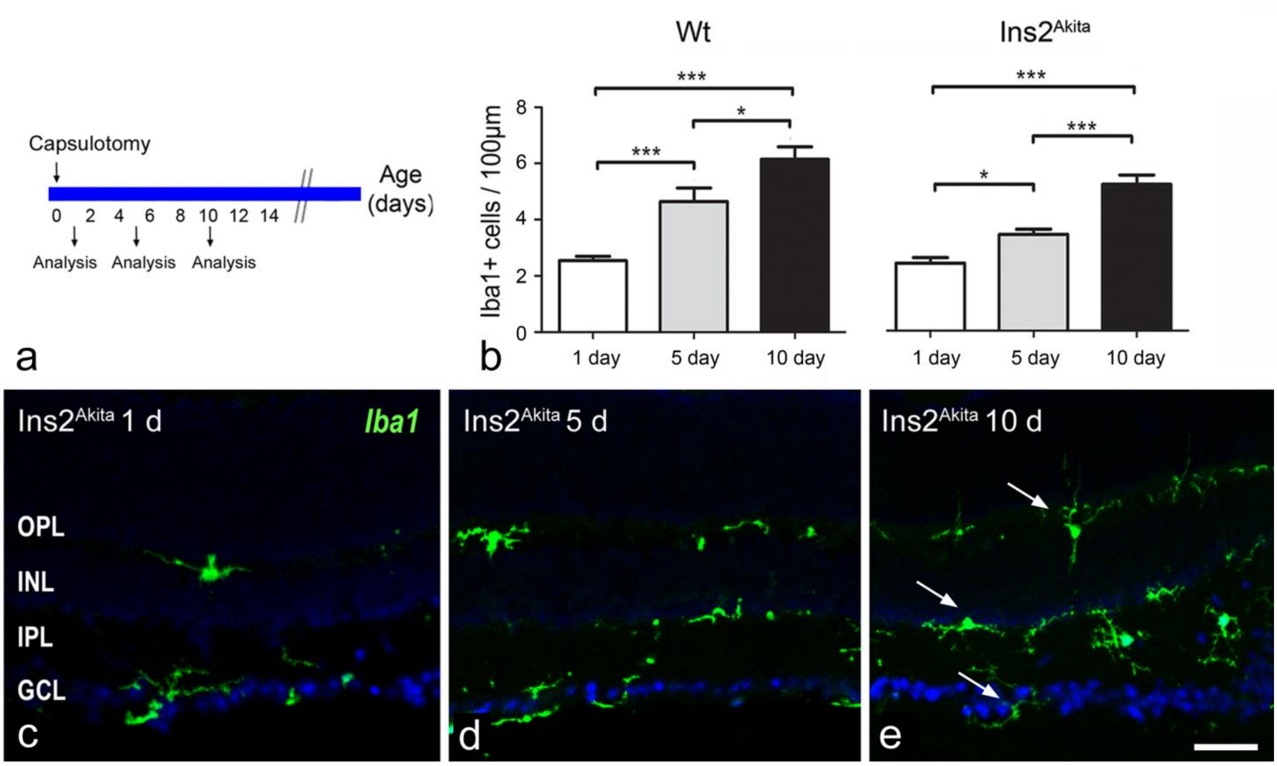


**ESM Figure 2. Time-dependent accumulation of retinal microglial cells following lens-capsulotomy.** (a) Lens-capsulotomy was performed in 3-month old WT and *Ins2$^{Akita}$* mice and eyes collected at different time-points post-surgery (1-, 5- and 10-days) and processed for Iba-1 immunostaining. (b) The density of Iba1$^{+}$ microglial retinal cells in different treatment groups. (c-e) Iba1$^{+}$ microglial cells progressive accumulate through all retinal layers (e, arrows) after lens-capsulotomy. n = 9 retinal sections per strain/condition/time-point. Results are presented as mean ± SEM. *$p < 0.05$, **$p < 0.01$, ***$p < 0.001$ compared between different groups. One-way ANOVA. OPL, outer plexiform layer; INL, inner nuclear layer; IPL, inner plexiform layer; GCL, ganglion cell layer. Scale bar = 30µm

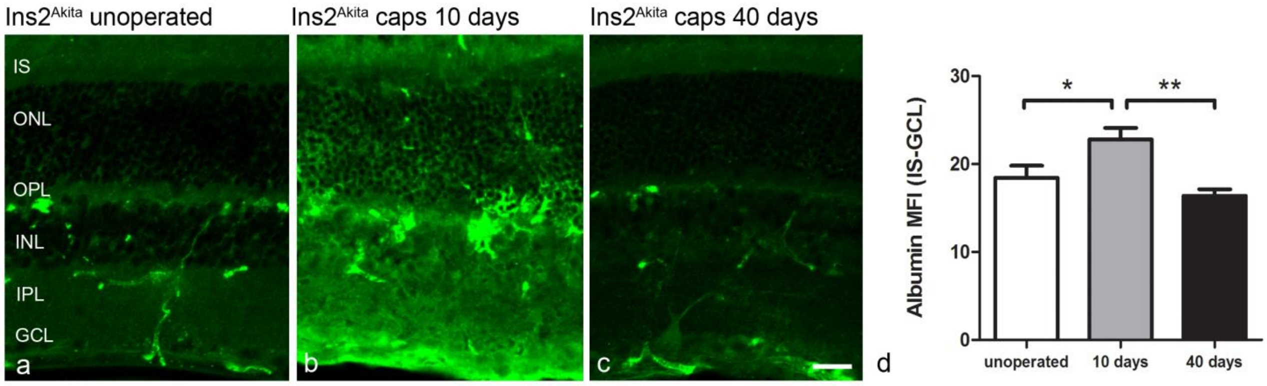


**ESM Figure 3. Blood retinal barrier breakdown in Ins2$^{Akita}$ mice shortly after capsulotomy.** (a-c) Lens-capsulotomy was performed in 3-month *Ins2$^{Akita}$* mice and eyes collected at different time-points post-surgery (10- and 40-days) and processed for albumin immunostaining. (d) The mean fluorescence intensity (MFI) of albumin between the inner segments (IS) of photoreceptors and ganglion cell layer (GCL) in different treatment groups. n = 20 retinal images per time-point. Results are presented as mean ± SEM. *$p < 0.05$, **$p < 0.01$ compared between different groups. One-way ANOVA. ONL, outer nuclear layer; OPL, outer plexiform layer; INL, inner nuclear layer; IPL, inner plexiform layer. Scale bar = 30μm

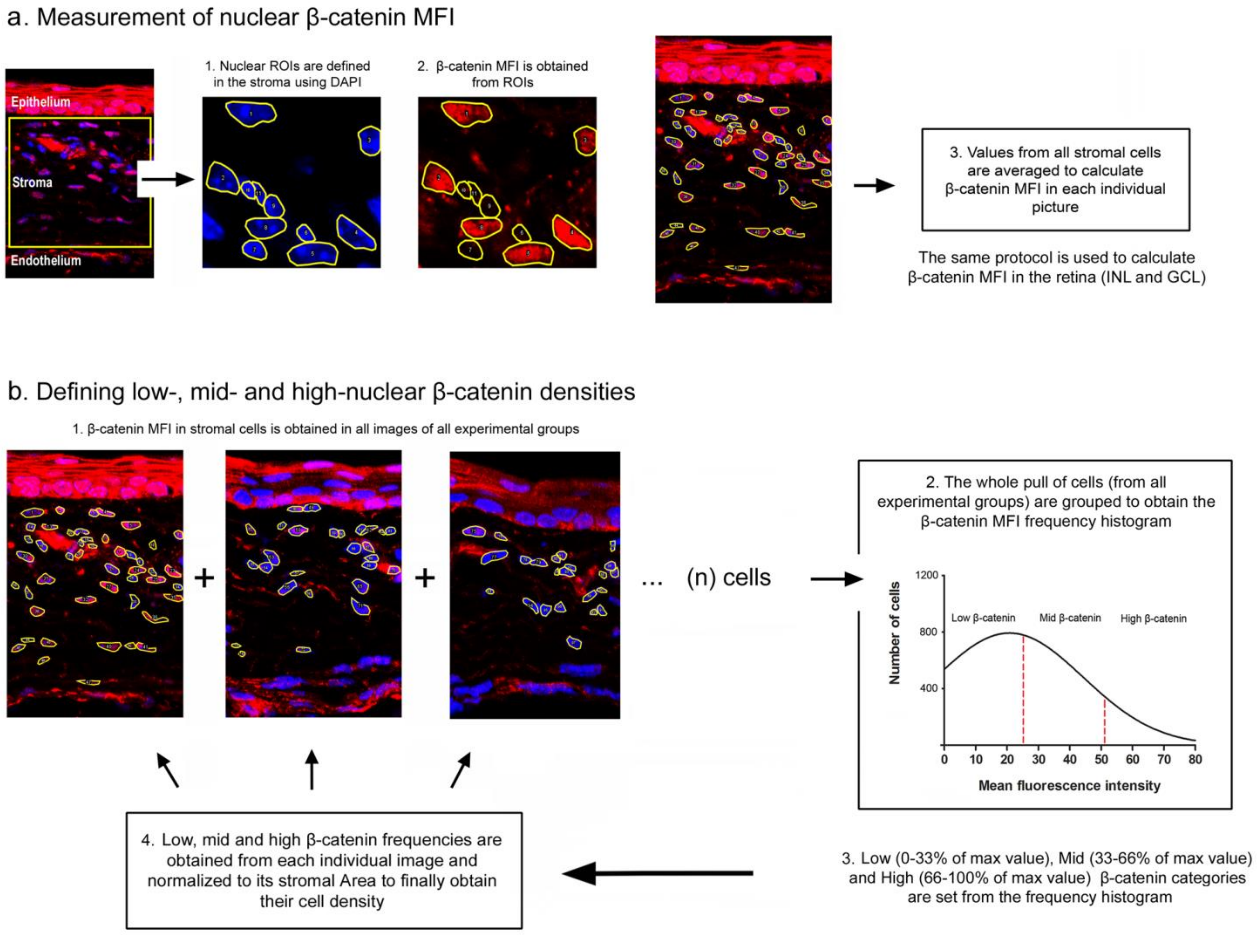


**ESM Figure 4. Experimental approach for measuring nuclear β-Catenin levels in eye tissues by immunohistochemistry.** (a) Mean fluorescence intensity (MFI) of β-Catenin in the nucleus of stromal cells at the irido-corneal junction and in retinal cells, were quantified by defining nuclear ROIs (region of interest) in a blind manner using DAPI staining. (b) To calculate the stromal cell densities of low-, mid- and high-nuclear β-catenin at the irido-corneal junction, we first obtained the MFI nuclear β-catenin values comprising all experimental groups. This pull of cells were the grouped to obtain the β-Catenin MFI frequency histogram, to define the low- (0 to 33% of max value), mid- (33 to 66% of max value) and high-β-catenin (66 to 100% max value) groups. Once defined, the frequencies of cells comprising these categories are obtained in each confocal photomicrograph, and their density obtained by normalization with the stromal area.

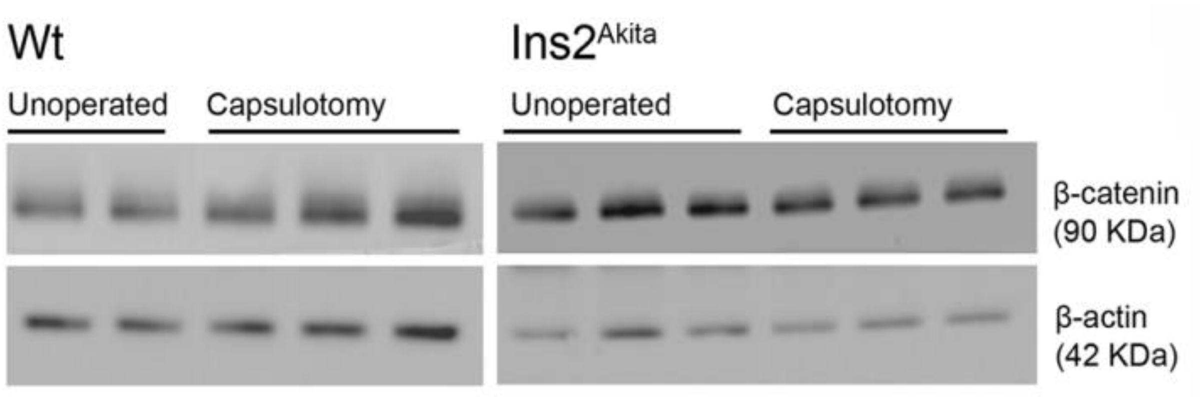


**ESM Figure 5. β-Catenin levels in the retina following lens-capsulotomy.** Lens-capsulotomy was performed in 3-month old WT and *Ins2*$^{Akita}$ mice and protein collected from retina 10 days after capsulotomy for western blot analysis of β-Catenin. β-actin was used for loading house-keeping control.

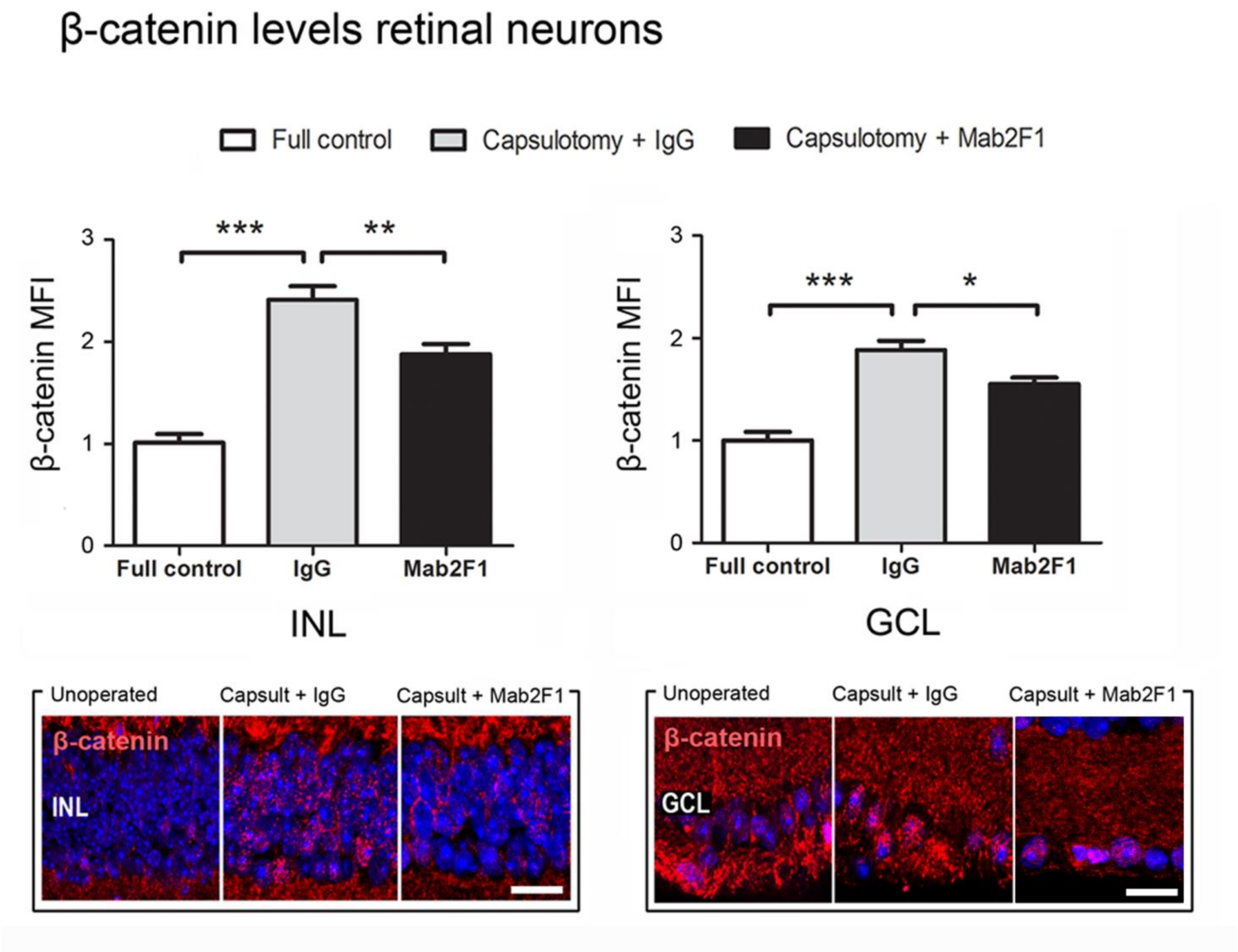


**ESM Figure 6. Mab2F1 downregulates nuclear contents of β-Catenin in the neuroretina of Ins2$^{Akita}$ mice after capsulotomy.** The mean fluorescence intensity (MFI) of β-catenin in the nucleus of inner nuclear layer (INL) and ganglion cell layer (GCL) of *Ins2$^{Akita}$* mice in different treatment groups. The lower panel shows representative confocal micrographs of β-catenin immunoreactivity among the different groups. n = 20 retinal images per strain/condition. Results are presented as mean ± SEM. *$p < 0.05$, **$p < 0.01$, ***$p < 0.001$ compared to unoperated or IgG treated mice of same strain. One-way ANOVA. INL, inner nuclear layer; GCL, ganglion cell layer. Scale bar = 20µm.
